# Developmental evolution of flowering plant pollen tube cell walls: callose synthase (*CalS*) gene expression patterns

**DOI:** 10.1186/2041-9139-2-14

**Published:** 2011-07-01

**Authors:** Jason M Abercrombie, Brian C O'Meara, Andrew R Moffatt, Joseph H Williams

**Affiliations:** 1 Department of Ecology and Evolutionary Biology, University of Tennessee, Knoxville, TN, USA

## Abstract

**Background:**

A number of innovations underlie the origin of rapid reproductive cycles in angiosperms. A critical early step involved the modification of an ancestrally short and slow-growing pollen tube for faster and longer distance transport of sperm to egg. Associated with this shift are the predominantly callose (1,3-β-glucan) walls and septae (callose plugs) of angiosperm pollen tubes. Callose synthesis is mediated by callose synthase (CalS). Of 12 *CalS *gene family members in *Arabidopsis*, only one (*CalS5*) has been directly linked to pollen tube callose. *CalS5 *orthologues are present in several monocot and eudicot genomes, but little is known about the evolutionary origin of *CalS5 *or what its ancestral function may have been.

**Results:**

We investigated expression of *CalS *in pollen and pollen tubes of selected non-flowering seed plants (gymnosperms) and angiosperms within lineages that diverged below the monocot/eudicot node. First, we determined the nearly full length coding sequence of a *CalS5 *orthologue from *Cabomba caroliniana *(*CcCalS5*) (Nymphaeales). Semi-quantitative RT-PCR demonstrated low *CcCalS5 *expression within several vegetative tissues, but strong expression in mature pollen. *CalS *transcripts were detected in pollen tubes of several species within Nymphaeales and Austrobaileyales, and comparative analyses with a phylogenetically diverse group of sequenced genomes indicated homology to *CalS5*. We also report *in silico *evidence of a putative *CalS5 *orthologue from *Amborella*. Among gymnosperms, *CalS5 *transcripts were recovered from germinating pollen of *Gnetum *and *Ginkgo*, but a novel *CalS *paralog was instead amplified from germinating pollen of *Pinus taeda*.

**Conclusion:**

The finding that CalS5 is the predominant callose synthase in pollen tubes of both early-diverging and model system angiosperms is an indicator of the homology of their novel callosic pollen tube walls and callose plugs. The data suggest that *CalS5 *had transient expression and pollen-specific functions in early seed plants and was then recruited to novel expression patterns and functions within pollen tube walls in an ancestor of extant angiosperms.

## Background

The pollen tube is a unique feature of male gametophytes of seed plants. In cycads and *Ginkgo*, pollen tubes are long-lived and function solely as haustorial, highly branched structures that grow invasively into female tissues [1-3]. In conifers and Gnetales pollen tubes function in a new way to deliver non-motile sperm to the egg (siphonogamy), while generally retaining a haustorial growth pattern [2,3]. Flowering plant (angiosperm) pollen tubes have lost most features of haustorial growth - their pollen tubes are typically short-lived and seem to function exclusively to deliver sperm to the egg [4,5]. The origin of siphonogamy has been held up as a classic example of exaptation [6], because the plesiomorphic function of the pollen tube - nutritional support for the male gametophyte - was subsequently co-opted for a novel role in sperm delivery [3]. Yet siphonogamy is clearly a complex process, and it is not at all obvious which aspects have common origins, which represent modifications of an ancestral pattern, and which have arisen independently in separate lineages [1,3,4,7]. Understanding the homologies of pollen tube structure and growth pattern may provide deeper insights into the origin(s) of this remarkable innovation.

Angiosperm pollen tubes have a unique wall structure. Their thin growing tip is comprised almost entirely of pectins. Just behind the pectic tip, cellulose synthases operate to form a very thin, pecto-cellulosic primary wall. Then, still in the subapical region, (1,3)-β-glucan (callose) is synthesized beneath the thin primary wall to form a thick layer [8]. The mature pollen tube wall of most angiosperms is primarily made of callose (81% by weight in *Nicotiana*; ref. 9). As an amorphous polysaccharide, callose can be synthesized more rapidly than an equivalent weight of fibrous cellulosic cell wall [9] and it provides resistance to tensile and compression stress [10]. Callose also severely reduces wall permeability and since angiosperm pollen tube walls are also prone to forming septae ("callose plugs") [11], the plesiomorphic haustorial function of tubes is largely precluded. These patterns are general features of all angiosperms, from *Amborella *and water lilies to *Arabidopsis *and maize [4,12]. Yet, despite their ubiquity, the ancestral function of callose walls and plugs is not obvious. Tubes that lack callose in their walls retain their function in some derived eudicot lineages, such as Lamiales [13] and in an *Arabidopsis *mutant line [14,15], though they have reduced competitive ability in the latter [14].

Pollen tubes in ovules of gymnosperms rarely contain callose in lateral walls, and callose plugs have never been reported [16]. Callose is found in the tip wall of growing pollen tubes of some conifers [17], a pattern never seen in angiosperms. Studies of *in vitro-*grown gymnosperm pollen tubes do sometimes find callose (or mixed-glucans) in lateral tube walls [17,18]. Importantly, the deposition of callose is generally transient in gymnosperm male gametophytes and its extent and location varies even among closely related species [17-20]. Such transient and variable phenotypes contrast with the relatively invariant and persistent expression pattern seen in angiosperm pollen tubes.

Callose synthesis is mediated by the enzyme, callose synthase, encoded by the callose synthase gene, and originally described as a glucan synthase-like gene (*GSL*) in *Nicotiana alata *[21]. Hong *et al*. (2001) named the callose synthase gene family *CalS *after identifying 12 gene family members in *Arabidopsis thaliana *[22]. *AtCalS5 *and its characterized orthologues (*NaGSL1 *from *N. alata*) have been directly linked to pollen tube wall formation and callose plug deposition, as well as to pollen exine development [14,15,23,24].

*CalS5 *appears to have an ancient origin by duplication. A comparative phylogenetic analysis of all *CalS *paralogs from the genomes of the moss, *Physcomitrella patens *[25] and *Arabidopsis *found that *AtCalS5 *was more closely related to a *Physcomitrella CalS *gene copy (*PpCalS5*) than to any other *Arabidopsis *paralog. Because callose was observed in the moss spore aperture region and *PpCalS5 *was identified as a putative orthologue to *AtCalS5*, PpCalS5 was hypothesized to play a role in moss spore germination [25]. If so, then *CalS5 *involvement in pollen tube growth may ultimately derive from a more ancient function involving the germination process. As such, changes in gene regulation were likely prerequisites for the acquisition of novel callose deposition patterns in angiosperm pollen tube walls. Alternatively, the patterns arose via duplication and functional divergence of a *CalS *gene within seed plants, or perhaps within the stem lineage leading to angiosperms.

In this paper we present molecular evidence that *CalS5 *orthologues are expressed in mature pollen and pollen tubes of several extant early-diverging angiosperms in Nymphaeales and Austrobaileyales and likely also in *Amborella trichopoda*. *CalS5 *orthologues are also expressed in mature gymnosperm pollen, including one siphonagam (*Gnetum*) and one non-siphonogam (*Ginkgo*). In the siphonogamous conifer, *Pinus*, we report a potentially unique *CalS *gene expressed in germinated pollen. We discuss the implications of these findings for the evolution of the angiosperm pollen tube wall and suggest new avenues of research to clarify the functional roles of *CalS *in the seed plant male gametophyte.

## Results

### Putative orthologues of *CalS5 *are expressed in pollen and pollen tubes of early-diverging angiosperms

**Figure 1:**
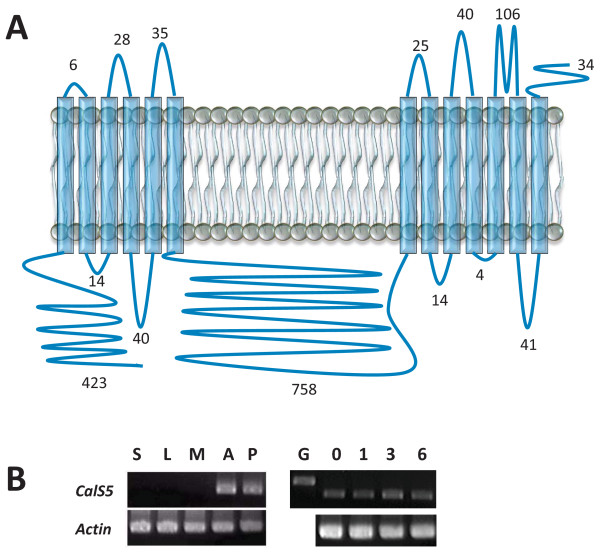
**Putative CalS5 orthologue (CcCalS5) topology and expression in *Cabomba caroliniana *and *Nymphaea odorata***. **A**, Predicted topology for CcCalS5 isolated from *Cabomba *pollen. Numbers indicate predicted amino acids in each domain. **B**, Semi-quantitative RT-PCR. Gels on left are from *Cabomba *cDNA derived from different tissues: S-stem tissue, L-leaf tissue, M-meristem tissue, A-pre-dehiscent anther and pollen, P-mature pollen grains. Gels on right are from *Nymphaea *cDNA: G-genomic DNA control, 0-mature pollen stage, 1, 3 and 6-pollen tubes at 1, 3 and 6 h post-inoculation.

A nearly full length coding sequence was obtained from *Cabomba caroliniana *(Cabombaceae; Nymphaeales) (*CcCalS5*) comprising 5,562 bp which translated into a predicted 1854 amino acid polypeptide with 78% identity to *Arabidopsis thaliana *CalS5 (AtCalS5) and 66% identity to the moss orthologue (PpCalS5) from the *Physcomitrella patens *genome [25]. The deduced polypeptide has a predicted topology containing between 13 and 17 transmembrane helices (as predicted by TMHMM v. 2.0; CBS, Lyngby, Denmark and SOSUI engine ver. 1.11; Nagoya University, Nagoya, Japan), a cytoplasmic N-terminal loop domain containing > 423 amino acids, and a large hydrophilic loop domain consisting of 758 amino acids, with loops between the helices ranging from 4 to 106 amino acids in length (Figure 1A).

RT-PCR was used to assess the presence of *CcCalS5 *transcript in various tissues of *C. caroliniana*, as well as in a pollen tube time-course experiment in *Nymphaea odorata *(Figure 1B) using intron-spanning primers. A 1,497 bp nucleotide sequence that shared 100% nucleotide identity with the *CcCalS5 *was amplified from *N. odorata *pollen and was used to designate the *Nymphaea CalS5 *orthologue (*NoCalS5*) in our RT-PCR experiments (Figure 1B). *CcCalS5 *expression was clearly observed in pre-dehiscent anthers and mature pollen of *Cabomba *(Figure 1B; left panel gel), but was also detected in low abundance in stem and leaf tissues during our RT-PCR optimization experiments (See Additional file 1). Expression of *CcCalS5 *in both *Cabomba *stem and leaf tissues was confirmed by sequencing clones of PCR products. *NoCalS5 *was consistently expressed during a time-course of *in vitro*-grown pollen tubes harvested at 0, 1, 3 and 6 hrs after inoculation in liquid medium (Figure 1B; right panel gel).

**Figure 2:**
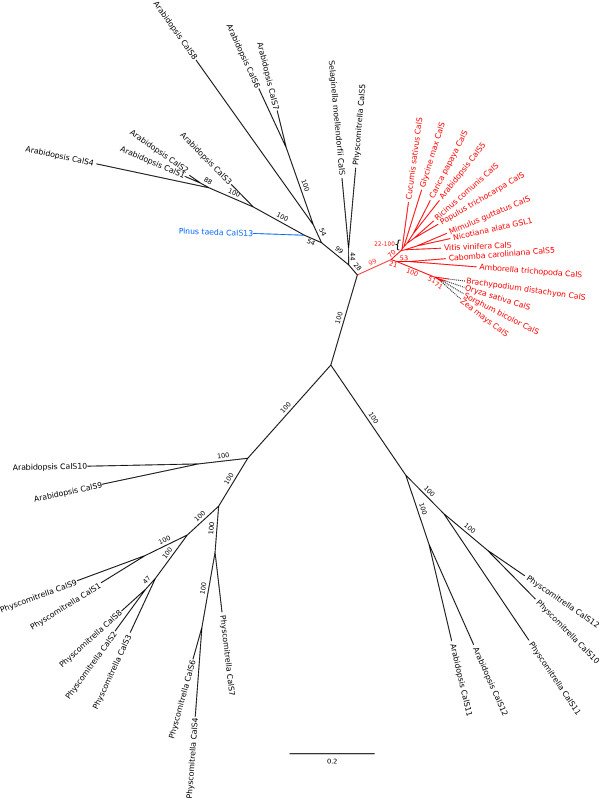
**Phylogenetic tree for full length CDSs of *Arabidopsis*, *Physcomitrella CalS *gene families and *CalS5 *orthologues**. Phylogenetic tree based upon alignment of predicted polypeptides for full length CDSs of *Arabidopsis*, *Physcomitrella CalS *genes, and putative *CalS *orthologues identified in this study. Sequences for *Amborella trichopoda *CalS5 and *Pinus taeda *CalS13 are from partial cDNA fragments containing 196 and 471 amino acids, respectively.

In the 18-taxon phylogenetic tree constructed to infer orthologous relationships, all putative angiosperm *CalS5 *orthologues plus *CcCalS5*, formed a clade with 99% bootstrap support (Figure 2). A partial sequence from the putative *CalS5 *orthologue in *Amborella trichopoda *[26] also falls within the *CalS5 *clade (Figure 2). The putative *CalS5 *orthologues of *Physcomitrella *[25] and *Selaginella *(this study) are strongly supported as falling in an angiosperm clade of *CalS *paralogs, but not necessarily as sister to the *CalS5 *clade.

RT-PCR also recovered *CalS *transcripts from the mature pollen of the early-diverging angiosperms, *Austrobaileya scandens *(Austrobaileyaceae; Austrobaileyales), *Nuphar advena *(Nymphaeaceae; Nymphaeales), and *Trithuria austinensis *(Hydatellaceae; Nymphaeales). These partial sequences from the hydrophilic loop domain align with the partial *NoCalS5 *sequence and are orthologous to *CalS5*, based on phylogenetic analysis (Additional files 2 and 3).

### Putative orthologues of *CalS5 *are expressed in pollen of *Gnetum gnemon *and *Ginkgo biloba*

**Figure 3:**
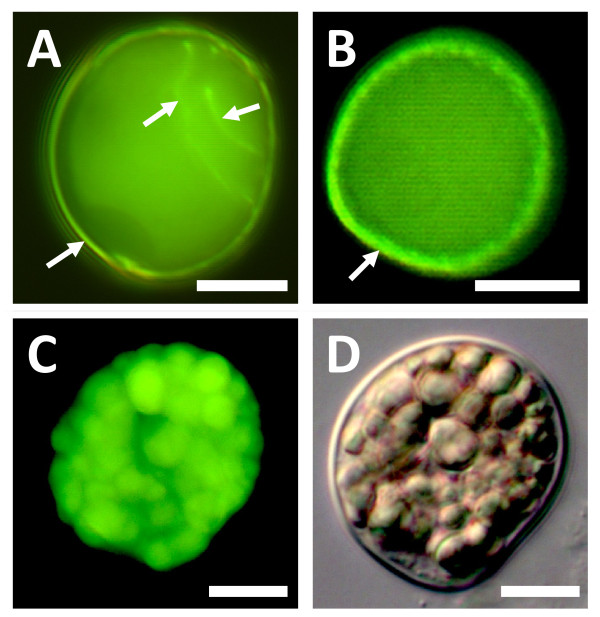
**Cell wall staining in *Ginkgo biloba *and *Gnetum gnemon***. **A**, *Ginkgo *pollen grain showing aniline blue staining of internal male gametophyte walls and intine (arrows; one day of *in vitro *growth). **B**, Aniline blue stain localized to inner wall of *Gnetum *pollen (arrow) before exine shedding (one day of *in vitro *growth). **C, D**, *Gnetum *pollen grain after exine shedding (eight days of *in vitro *growth). C, Lack of aniline blue staining of intine (compare with DIC view in D). Note pollen is larger than first-day pollen in B and now contains abundant starch grains. Scale bars, 10 μm.

At 24 h after incubation, *Ginkgo *pollen stained for callose in the aperture area, intine, and also in the walls that separated prothallial, generative, and tube cells (Figure 3A). *Gnetum *pollen sheds its exine before tube growth, and prior to exine shedding, aniline blue staining was observed in the inner pollen wall (Figure 3B). After exine shedding, callose was not observed in the intine (Figure 3C, D).

Partial cDNA fragments from putative *CalS5 *orthologues were amplified from mature pollen of *Gnetum gnemon *(*GgCalS5*) and *Ginkgo biloba *(*GbCalS5*). Their predicted amino acid sequences aligned with the central loop domains of other CalS proteins, including those known to function during *Arabidopsis *pollen development (see Additional file 2). Phylogenetic analysis placed the *Ginkgo *and *Gnetum *sequences within a strongly supported clade of angiosperm CalS5 sequences (Additional file 3).

Interestingly, the aligned predicted protein sequences identified a short NASQ motif that *Ginkgo *shares with all members of Nymphaeales but that is absent from all other CalS5 sequences (see Additional file 2). Prosite scans of the aligned sequences identified the shared motif as a putative N-glycosylation site (Prosite scan data not shown). Other putative functional motifs common within this alignment are CK2 and PKC phosphorylation sites that are highly conserved within all other *Arabidopsis *CalS sequences, however one predicted cAMP-dependent phosphorylation site, K(R/K)ES, was unique to most taxa within the CalS5 clade (see Additional file 2).

### A unique *CalS *orthologue is expressed in *Pinus taeda *pollen

**Figure 4:**
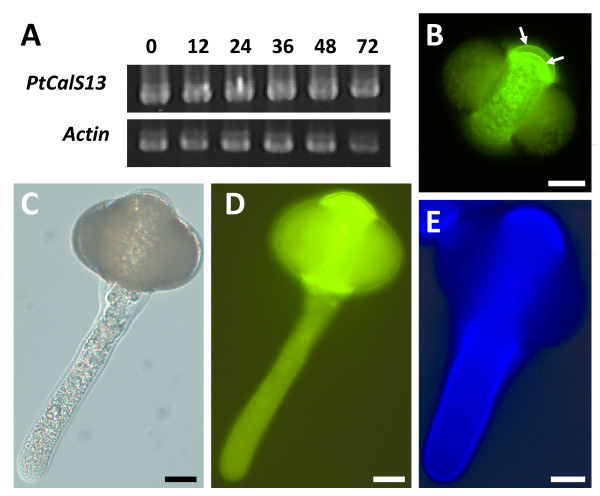
**Cell wall staining and *CalS *RT-PCR in *Pinus taeda *pollen tubes grown *in vitro***. **A**, Semi-quantitative RT-PCR of *PtCalS13 *from 0 to 72 h on growth medium. **B**, Ungerminated *P. taeda *pollen after 12 h on growth medium. Note the weak band stained by aniline blue in distal aperture area (at bottom) and strong staining of prothallial cell walls on proximal side of pollen grain (white arrows). **C-E**, Pollen tube after 48 h on growth medium (24 h after germination). In D, note strong aniline blue staining at the distal aperture area, and lack of stain in tube walls (compare with C). Calcofluor localizes to both intine and tube wall (E). Scale bars, 20 μm.

A 1,413 bp *CalS *transcript was strongly expressed in mature and germinated *Pinus taeda *pollen (Figure 4A). Phylogenetic analysis of the 471 amino acid predicted polypeptide indicates 99% bootstrap support for its inclusion within a clade that does not include CalS5 (Figure 2). Because it is distantly related to any of the known *Arabidopsis *paralogs in that clade, we named the gene *PtCalS13*. *PtCalS13 *transcripts were abundant over a 72-hour time period of *in vitro *growth (Figure 4A), however repeated attempts to amplify *CalS5*-like transcripts over the same developmental stages failed. All combinations of primers that amplified *CalS5*-like fragments from *Ginkgo, Gnetum*, and early-divergent angiosperm pollen cDNA were attempted.

Aniline blue staining was localized to the inner walls of the mature pollen grain, but not the tube wall (Figure 4B-D). In pollen, strong staining was observed within the third intine layer at the proximal face of the microgametophyte, whereas weaker staining was apparent in the aperture region, the leptoma (Figure 4B). Upon germination, the aperture area became strongly stained, but the thick lateral walls of the pollen tube did not (Figure 4C, D). Calcofluor staining for cellulose was strong throughout all time periods observed during *in vitro *pollen tube growth (Figure 4E).

### Comparative analysis of predicted functional motifs among callose synthases

Protein motif searches [27,28] were performed on the sequences of CalS5 orthologues, as well as on all other callose synthases in *Physcomitrella *and *Arabidopsis *to evaluate conserved patterns of short linear motifs that may have functional significance. Comparison of the callose synthase protein sequences revealed conservation of seven different site patterns in all callose synthases: N-glycosylation, cAMP- and cGMP-dependent protein kinase phosphorylation, protein kinase C phosphorylation (PKC), casein kinase II phosphorylation (CK2), tyrosine kinase phosphorylation, N-myristoylation, and amidation (data not shown). The majority of putative functional motifs were found within the N-terminal loop and the large central loop domain. The central loop domains of all callose synthases were consistently enriched with predicted PKC and CK2 phosphorylation sites. There were no clear distinguishing features of CalS5 orthologues with respect to selected patterns of predicted linear motifs when compared to the other callose synthases. *Zea mays*, *Sorghum bicolor*, and *Selaginella moellendorfii *all appear to have truncated CalS5 proteins, with the entire N-terminal domains completely absent. This may reflect incorrect annotation, given the conserved nature of this large functional domain.

### *In silico *identification and phylogenetic analysis of putative CalS5 orthologues

BLAST searches of NCBI [29] and Phytozome [30] databases revealed putative *CalS5 *orthologues from a phylogenetically diverse group of plant species which ranged from the unicellular green algae, *Chlamydomonas reinhardtii *to most completely sequenced angiosperms. Amino acid sequences from these putative orthologues were aligned with all known callose synthase gene family members from *Physcomitrella*, *Arabidopsis*, as well as other putative *CalS5 *orthologues for other angiosperms and the spikemoss, *Selaginella moellendorfii*. Also included in the alignment were two sequences of particular interest, the 471 amino acid PtCalS13 sequence obtained from *Pinus taeda *pollen cDNA, and a putative *CalS5 *orthologue identified *in silico *from the early-diverging angiosperm, *Amborella trichopoda*. The *Amborella *translated uniscript was obtained from the 454-EST build from the Ancestral Angiosperm Genome Project website [26] and comprised 196 amino acids. Several other translated uniscripts from *Amborella *displayed homology to CalS5, but because contig assembly was not feasible, they were not included in this analysis (data not shown).

## Discussion

### CcCalS5 contains both general CalS functional motifs and CalS5-specific motifs

All CalS proteins studied to date share a common topology with a large N-terminal hydrophilic domain followed by two clusters of transmembrane (TM) domains that flank a large central hydrophilic domain [15,22,23,31,32]. The large hydrophilic loop is thought to accommodate interactions with other proteins, such as Rop1, UDP-glucose transferase (UGT1), sucrose synthase, and annexin, which enable the formation of a CalS enzyme complex [31]. Scans for functional motifs in CcCalS5 support the model of Verma and Hong [31], in which most known callose synthase proteins exhibit similarities in putative glycosylation and phosphorylation sites, particularly within the N-terminal domain and the large central loop.

With respect to pollen tube growth, cAMP is a known signalling molecule for pollen tube guidance and growth [33]. In our alignment of the loop domains, we identified one putative cAMP and cGMP-dependent phosphorylation site common to all seed plant members of the CalS5 clade, but absent from PpCalS5 of *Physcomitrella *and from all other CalS paralogs (see Additional file 2). We also identified a putative CalS5 N-glycosylation site unique to seed plants - present in *Ginkgo *and all members of the Nymphaeales clade (see Additional file 2) but absent from *Austrobaileya *and all monocots and eudicots. It is not known whether these variants cause functional differences in *CalS5 *expression.

### *CalS *genes involved in male gametophyte development

Five of 12 callose synthase genes (*CalS5*, *CalS9*, *CalS10*, *CalS11*, and *CalS12*) have been shown to function during microsporogenesis and microgametogenesis in *Arabidopsis *[15,34-37]. In contrast to other *CalS *genes, *CalS11 *and *CalS12 *contain only two or three exons [22] and are Ca^2+^-dependent [38]. *CalS11 *and *CalS12 *are genetically linked and perform partially redundant roles in the formation of walls separating microspores of the tetrad and in late maturation of the male gametophyte [34]. *CalS12 *is also activated during wound response and on stigmatic papillae [39].

The other 10 *CalS *genes contain between 39 and 50 exons and are Ca^2+^-independent [40,41]. *CalS5 *is sporophytically expressed to form the callose wall of pollen mother cells and microspore tetrads and is also the predominant gametophytically-expressed transcript in germinating pollen and growing pollen tubes [14, 15, this study]. *CalS9 *and *CalS10 *function early in microgametogenesis [35,36] since mutant lines independently exhibited functional aberrations during the entry of microspores into mitosis. Mutants of *CalS9 *caused failure of mitosis II as well as abnormal positioning of nuclei in mature pollen and precocious germination, inside the anther [37]. *CalS10 *is known to be involved in cell plate formation [22]. Silencing of *CalS9 *and *CalS10 *using gene-specific dsRNAi constructs also resulted in a dwarfed growth habit, suggesting that both also function in the sporophytic phase [35]. These studies show that male gametophyte development in *Arabidopsis *is mediated by transient expression of a number of *CalS *genes, whereas in all angiosperms studied to date *CalS5 *is abundant and predominant during pollen germination and in all stages of pollen tube growth.

### The *Pinus taeda *male gametophyte expresses a unique *CalS *gene in pollen

We found strong expression of a novel *CalS *gene in mature and in germinated pollen of *P. taeda*. *PtCalS13 *was strongly supported as falling within a clade of *AtCalS *paralogs that does not include any of the known gametophytically-expressed paralogs (*AtCalS5 *and *AtCalS9-12*). *PtCalS13 *cannot be a deeply divergent copy of *CalS5 *or *CalS9-12*, but is most likely a novel copy of *CalS *(alternatively, it may be a deeply divergent orthologue of *CalS1-4 *or *CalS6-8*; Figure 2). *CalS5 *transcripts were not found at the same stages of development, suggesting *PtCalS13 *functions in place of *CalS5*. However, more work is needed to test this hypothesis.

*Pinus *species are quite variable in the location and extent of pollen callose deposition [42,43]. In *P. taeda*, callose was present in the intine and male gametophyte walls before pollen germination. Upon germination, it became strongly expressed in the aperture region surrounding the exiting pollen tube, but did not extend into the tube wall, as it does in angiosperms [41,44,45]. Thus, the callose distribution pattern associated with *PtCalS13 *expression is quite different from the *CalS5 *pattern in germinating angiosperm pollen.

### *Ginkgo *and *Gnetum *express an orthologue of *CalS5 *in pollen

There is strong evidence that the *CalS *genes expressed in mature pollen of *Gnetum *and *Ginkgo *are orthologous to angiosperm *CalS5 *since their protein sequences were nested within the angiosperm CalS5 clade. Callose deposition is apparently restricted to pollen in these species. In *Gnetum *it was present in the inner wall of pollen before exine shedding, but after shedding it was absent from the intine which is continuous with the emerging pollen tube wall. In *Ginkgo*, callose was abundant in the intine and in the walls of the male gametophyte. A study of *in vitro *pollen tube growth in *Ginkgo *found that their tube walls stained weakly for aniline blue but reacted strongly to calcofluor white and β-(1,3)(1,4) antibodies, suggesting the presence of β-(1,3)(1,4) mixed glucans [18].

### The evolutionary developmental origins of callose synthase expression in angiosperm pollen tubes

**Figure 5:**
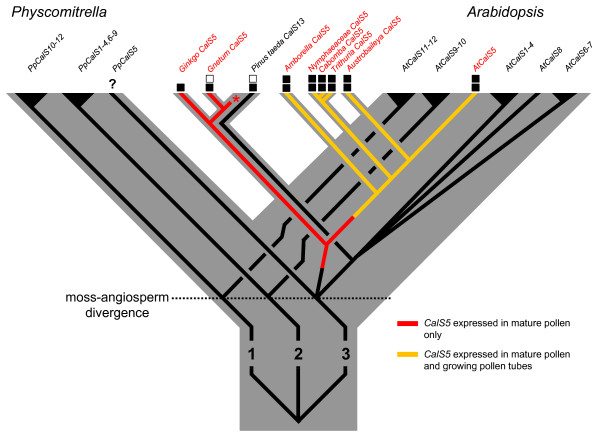
**Hypothesized evolution of *CalS5 *gene expression in seed plant pollen**. *CalS *gene relationships from Figure 2 and Additional file 3 are superimposed on a Moss (*Physcomitrella*) + Seed plant species tree [71]. Filled boxes indicate aniline blue staining of the inner wall of mature pollen (lower) or pollen tube walls/callose plugs (upper) and have been associated with *CalS5 *or *CalS13 *expression in all but *Amborella *(see text). Unfilled boxes represent lack of staining for callose. Branches are coloured to reflect inferred changes in *CalS5 *gene expression patterns, based on data from this study. Nymphaeaceae includes *Nymphaea *and *Nuphar*.

Fast growing pollen tubes are arbiters of the rapid reproductive cycles of angiosperms and their unique wall structure may have been a trigger for extensive pollen tube growth rate evolution in the group [1,4,46]. Gene family expansions are thought to have been important for trait diversification early in angiosperm history or pre-history [47,48]. Six *CalS *paralogs from three ancient land plant lineages - five from *Arabidopsis *[22] and one from *Pinus *(this study) - are now known to be expressed in male gametophytes of seed plants (Figure 5). To date, only *CalS5 *has been shown to be expressed in pollen tubes, and only in a few model system eudicots [15,23,24]. The finding that pollen tubes of a broad set of extant early-diverging angiosperms also utilize *CalS5 *in their pollen tubes supports the homology of callose walls and plugs in flowering plants [4,5].

*CalS5 *transcripts were also found in mature and/or germinating pollen, of two distantly-related gymnosperms, *Ginkgo *and *Gnetum*. The predominant anatomical location of callose in *Ginkgo *was in the intine and internal gametophytic cell walls, whereas the pollen tube-forming intine of germinated *Gnetum *pollen did not contain callose. In all early-divergent angiosperms used in this study, callose was present within the intine of germinating pollen and continuous with the callose inner wall of the pollen tube [4,43,49]. Gametophytic expression of *CalS5 *has a similar pattern in *Arabidopsis *and tobacco [14,15,23,24,32,37,50]. Thus, it seems likely that *CalS5 *had an ancestral expression pattern within the inner pollen wall that later became modified via the evolution of gene regulation to function in growing pollen tubes of an ancestor of extant angiosperms (Figure 5).

A number of hypotheses have been proposed as to what the ancestral function of callose in germinating pollen or spores might have been. In the moss, *Physcomitrella*, callose was deposited in the inner exine layer (not the intine) near the aperture at the proximal pole of the spore just before germination [25]. Callose was inferred to function in spore germination and it was suggested that a *CalS5 *orthologue was involved [25]. Expression data are needed to determine if these results reflect an ancient aspect of land plant spore germination or an apomorphic feature of moss spores (Figure 5). We also found callose thickenings in the aperture area of germinating *Pinus *pollen, but these were associated with *PtCalS13*, not a *CalS5 *orthologue (Figure 5). Callose is localized to the inner intine of mature pollen of some *Pinus *species [51-53], the outer layer in *P. sylvestris *[17,54], and is absent from the intine of *P. wilsonii *[55]. Pacini *et al*. (1999) concluded that such variable and transient expression of callose in *Pinus *pollen indicates that it functions as a reserve polysaccharide, rather than serving a structural or prophylactic function [45]. Górska-Brylass (1970) argued its presence in the proximal outer intine was due to non-retrieval of callose plates from the prior divisions of prothallial cells [56]. Alternatively, its co-localization with degenerate prothallial cells may indicate the prior involvement of callose as a wall sealant that initiates cell death, a pattern also seen during megasporogenesis in seed plants [57].

To date no study has convincingly shown callose to be the predominant and permanent constituent of any gymnosperm pollen tube wall, nor is there any finding of a callose plug in a gymnosperm pollen tube. For example, callose is reported from young but not old tubes in *Pinus *and *Cycas *[17,20]; it is a transient feature of long-lived *Pinus *tubes during winter dormancy [17]; and it can appear at the growing tube tip but does not persist in the lateral walls as the tip continues its growth [16,17]. From a functional standpoint, one should note that the origin of persistent callose walls and plugs in angiosperm pollen tubes must have been contingent on a shift from a primarily haustorial function, such as occurs in root hairs, rhizoids and pollen tubes of Cycads and *Ginkgo*, to a new one in which a short-lived and fast-growing tip functions primarily to carry sperm to egg. Pollen tubes of gymnosperms are long-lived and extend from a multicellular male gametophyte, which typically lives within a pollen grain attached to female tissues at the pollination site [3]. In most conifers, sperm are formed late in male gametophyte ontogeny and must travel from the pollen grain to the tip late in life. Thus, the biology of the fertilization process in most gymnosperms prevents their pollen tubes from utilizing callose as a semi-permanent structural feature of their walls.

At some point(s) along the lineage leading to angiosperms, two shifts in CalS5 localization occurred - callose deposition became restricted to a short subapical region of the growing pollen tube tip and to a small distal region of the tube where a callose plug forms. Importantly, both of these changes in localization must also have involved changes in callose retrieval, giving rise to persistent callose walls and plugs. One model for the origin of angiosperm pollen tube morphology is that an ancestral conifer-like pollen tube became transformed by the evolution of faster growth rates [46], causing the callose synthesis and retrieval machinery to be displaced from the newly-forming tube tip to a subapical position [1,17]. Comparative analyses support the notion that early angiosperm pollen tubes grew faster than those of their gymnosperm-like ancestor (extant gymnosperm pollen tubes are characterized by exceptionally slow pollen tube growth rates) [5]. What is not clear is whether non-retrieval of callose is a cause or a consequence of the origin of faster growth rates.

Given the increased tendency of molecular phylogenetic analyses to place conifers/Gnetales in an isolated position relative to angiosperms [58], it is worth considering that angiosperm pollen tube morphology may have evolved independently from an ancestral *Ginkgo/*Cycad-like haustorial tube [20], rather than from a transitional siphonogamous, conifer-like predecessor. Resolving the question of pollen tube origins will require careful developmental analyses of pollen tube growth and the many genes that mediate differences among extant seed plant groups. It would be especially interesting to look at the evolution of the callase (β-1,3-glucanase) gene family, which catalyzes the retrieval of callose [59].

## Conclusion

This study supports the homology of callose pollen tube walls and plugs across flowering plants at the level of *CalS5 *gene expression. Since *CalS5 *was also found to be actively transcribed in mature pollen of *Ginkgo *and *Gnetum*, we suggest that CalS5 localization to the inner intine of mature germinating pollen was present in a distant angiosperm ancestor, perhaps extending into the walls of young pollen tubes, and was later co-opted as a non-transient feature of angiosperm pollen tubes (Figure 5). *CalS5 *is a structural gene that originated by duplication long before the origin of extant angiosperms. Thus, the novel callose deposition patterns of angiosperm pollen tubes must be a consequence of the evolution of novel regulation of an ancient gene. It remains to be seen to what extent this involved duplication and divergence of other genes involved in the callose synthesis or retrieval pathways, and to what degree it was or was not a developmental outcome of the evolution of faster growth rates.

## Methods

### Plant material

Whole flowers from *Austrobaileya scandens *White were collected near Millaa Millaa, Queensland, Australia (17° 31' 15" S, 145° 33' 53" E). *A. scandens *pollen tubes were grown in hanging drops of BK media [60] containing 2.5% sucrose inside closed petri plates for 5 to 12 hours. Pollen from *Trithuria austinensis *Sokoloff was collected in Branchinella Lake, shire of Manjimup, Western Australia (34° 16' S, 116° 42' E). Pollen or pollen tubes of these two species were centrifuged briefly and resuspended in RN *Later *(Ambion, Austin, TX, USA) and RNA was isolated within several weeks. RNA was isolated from fresh pollen or pollen tubes for the remaining species below. Pollen from *Ginkgo biloba *L. was collected from trees growing on the University of Tennessee campus in Knoxville, TN, USA. *Nuphar advena *Aiton. flowers were collected near Sparta, TN, USA (35° 55' 11" N, 85° 20' 41" W) and pollen tubes were grown in BK media with 5% sucrose for two hours before RNA isolation. *Nymphaea odorata *Aiton. flowers were collected from a pond in Knoxville, TN, USA (35° 53' 51" N, 84° 10' 23" W). *Cabomba caroliniana *A. Gray plants were grown in greenhouse water tanks and were originally collected from Racoon Creek, Jackson County, AL, USA (34° 46' N, 85° 50' W) or purchased from Carolina Biological Supply (Burlington, NC, USA). *Gnetum gnemon *L. flowers with dehiscent anthers were collected from greenhouse-grown plants in DEPC-treated water, vortexed to separate pollen from all other flower parts, and briefly centrifuged prior to RNA isolation. Pollen from *Pinus taeda *L. and *P. strobus *L. was collected from trees on the University of Tennessee campus. *Pinus *pollen was grown in a liquid medium containing 10% sucrose, 15 mM MES, 1 mM H_3_B, 1 mM CaCl_2_, pH 4.0 in petri plates sealed with parafilm.

### *In vitro *pollen tube experiments with *Nymphaea *and *Cabomba*

Due to the poor germination observed for both *Nymphaea *and *Cabomba *pollen in standard BK media, stigmatic fluid from first day *Nymphaea *flowers was collected on site with a disposable pipette and used as the pollen tube growth medium for these species. Both species exhibited 70 to 90% germination success when grown in the fresh stigmatic fluid, and thus fresh stigmatic fluid was used for all pollen tube experiments described here. Stigmatic fluid was collected shortly after flower opening (9 to 10 am), and centrifuged for three minutes at 13,000 rpm to remove any contaminating debris and/or pollen grains. Anthers from *Cabomba *and *Nymphaea *were removed with forceps and placed into 1.7 ml tubes that contained 1 ml of stigmatic fluid. Anther number was used to standardize samples for pollen density during tube growth. Tubes were vortexed briefly to separate pollen grains from anthers, anthers were then removed, and contents were transferred to a small petri plate for pollen tube growth at room temperature. *Nymphaea *pollen tubes were grown for various lengths of time (one, three, and six hours post-inoculation) for gene expression experiments. For RNA extractions, pooled pollen tube samples were harvested at each time point, centrifuged at a 2,000 rpm for 30 seconds to maintain pollen tube integrity, and immediately frozen in liquid nitrogen after growth medium was removed.

### Bioinformatics and primer design

In order to search for orthologous *CalS5 *gene sequence in the early-diverging angiosperms, the local BLAST tool on the ancestral angiosperm genome project website [26] was used to blast the *Arabidopsis *CalS5 protein sequence (tBlastn) against all available 454-Sanger hybrid databases. After performing non-redundant nucleotide NCBI BLAST searches of individual uniscript hits, primers were designed to amplify a *Nuphar advena *uniscript sequence (c78546) with the lowest E-value corresponding to *AtCalS5*. The primers designed towards *Nuphar advena *sequence (Sec16F; Sec17R) amplified a 600 bp highly conserved region of sequence within the predicted hydrophilic domain of the putative *CalS5 *orthologue. These primers were also used for amplifying putative *CalS5 *orthologous pollen-derived cDNA from *Ginkgo biloba*, *Austrobaileya scandens*, and the more closely related water lily species, *Cabomba caroliniana*, *Nymphaea odorata*, and *Trithuria austinensis*. Another forward primer (Sec17F) nested within this sequence and the reverse primer (Sec17R) enabled PCR amplification of the 250 bp *Gnetum *sequence. Various multiple sequence alignments were carried out on all callose synthase DNA and protein sequences in *Physcomitrella patens *and *Arabidopsis *to aid in primer selection. To amplify the *Pinus taeda CalS *cDNA, two EST sequences showing the highest homology to *AtCalS5 *and *PpCalS5 *sequence (AI812992 and FJ114840) obtained from non-redundant NCBI BLAST of *Pinus *were aligned with *PpCalS5 *and *AtCalS5*. Forward FJ114840-F and reverse primer AI812992-R amplified a product that reflected the alignment. To prevent amplification of other pollen-expressed *CalS *genes (*CalS9*, *CalS10*, *CalS11*, *CalS12*), particular attention was given to these sequences during primer design for PCR applications. All primer design and sequence alignments were performed using Vector NTI software (Invitrogen, Carlsbad, CA, USA). A complete set of primers used in this study is listed in Additional file 4.

### Molecular analyses

Total RNA from pollen and pollen tubes was isolated using Tri Reagent^J ^(Ambion, Austin, TX, USA) and a modified CTAB protocol was required for *Cabomba *vegetative tissues [61]. For cDNA synthesis, 1.5 μg of total RNA was used, according to the manufacturer's protocol (ArrayScript; Ambion, Austin, TX, USA). Prior to cDNA synthesis, all samples were subjected to DNase I (TurboFree DNase kit; Ambion). RNA was assessed for quality with agarose gel electrophoresis and quantified with a NanoDrop (Thermo Scientific, Waltham, MA, USA) spectrophotometer. A 3' RACE procedure was used according to the First Choice RLM RACE kit (Ambion, Austin, TX, USA) to acquire the 3' end of the *Cabomba caroliniana CalS5 *gene. However, due to difficulties with the 5' RACE protocol 5' products were amplified using a forward primer designed towards highly conserved sequence within a multiple sequence alignment of CalS5 orthologues (CalS515F). Inverse PCR was performed to obtain the 5' end of the full length cDNA using a digest/re-ligation/digest strategy with HindIII/PstI, respectively [62]. Although attempts to obtain the 5' end of the cDNA were incomplete, the inverse PCR procedure did enable the sequencing of introns that flanked the 5'-most exon. Identification of these intron-exon boundaries enabled the design of intron-spanning primers for semi-quantitative RT-PCR. All PCR reactions that required cloning were performed with Herculase II DNA fusion polymerase (Agilent, Santa Clara, CA, USA). Products were gel purified using a Qiaex II gel purification kit (Qiagen, Valencia, CA), cloned into pcr8-GW-TOPO (Invitrogen, Carlsbad, CA, USA), and sequenced on an ABI 3100 capillary sequencer at the University of Tennessee Molecular Biology Resource Facility. cDNA tissue sources are listed in Additional file 5. All sequences were deposited in Genbank [http://www.ncbi.nlm.nih.gov/genbank/index.html].

### Semi-quantitative RT-PCR

To prevent amplification of possible orthologous pollen-expressed *CalS *genes (*CalS9*, *CalS10*, *CalS11*, *CalS12*), both protein and nucleotide alignments were used to design primers to amplify a 631 bp intron-flanking sequence that shared a predicted low homology to non-target *CalS *cDNA. Primers were designed to amplify an *Actin *gene isolated from *Nuphar advena *pollen. *Actin *was also used as a reference gene to confirm equal template loading in *Pinus taeda *RT-PCR. A master mix of PCR reagents (described above) was used to amplify the *CalS5 *fragment and *Actin *control gene in separate reactions at equal template concentrations and cycling parameters. Threshold cycle optimization was determined by performing PCR amplifications over a range of cycles. The number of PCR cycles selected corresponded to where the trend line exhibited the highest correlation to exponential amplification. Bands representing each tissue type were purified, cloned, and the sequence was verified to confirm single product amplification.

### Phylogenetic analysis

Alignments of predicted amino acid sequences were performed using MAFFT (Cambridge, Engand) [63]. Mesquite (Vancouver, BC, Canada) [64] was used to truncate taxon names. Gblocks software (Barcelona, Spain) [65] with gap mode ALL was used to exclude poorly aligned regions. Model testing was performed using ProtTest 2.4 (Vigo, Spain) [66] using only those substitution models present in RAxML (San Diego, CA, USA) [67,68]. RAxML was used on the CIPRES web server [68,69] to infer phylogenetic trees and do fast bootstrapping (maximum likelihood method). Trees were visualized using FigTree (Edinburgh, Scotland, UK) [70].

## Abbreviations

AsCalS5: *Austrobaileya scandens *CalS5; CalS: callose synthase; CK2: casein kinase II; PKC: protein kinase C; CcCalS5: *Cabomba caroliniana *CalS5; GbCalS5: *Ginkgo biloba *CalS5; GgCalS5 *Gnetum gnemon *CalS5; GSL: glucan synthase-like; NoCalS5: *Nymphaea odorata *CalS5; PtCalS13: *Pinus taeda *CalS13; TaCalS5: *Trithuria austinensis *CalS5; UGT1: UDP-glucose transferase I.

## Competing interests

The authors declare that they have no competing interests.

## Authors' contributions

JA conceived of the study, carried out the experiments, and drafted the manuscript. AM performed troubleshooting of RNA isolation protocols and assisted in experiments. BO performed phylogenetic analyses and tree construction. JW also conceived of the study, provided experimental guidance, and shared in writing the manuscript. All authors read and approved the final manuscript.

## Supplementary Material

Additional fie 1 **N-terminal alignment of CcCalS5 with AtCalS5 and PpCalS5, and *CcCalS5 *expression in various tissues of *Cabomba caroliniana***. **A**) Amino acid alignment showing the expected missing sequence of the N-terminal end of the CcCalS5 cDNA. **B**) Agarose gel showing amplified PCR products that were cloned and sequenced to confirm the presence of *CcCalS5 *transcript in vegetative and reproductive tissues of *Cabomba*. S, stem tissue, L, leaf tissue, M, meristem tissue, A, pre-dehiscent anther, P, pollen from dehiscent anther. Click here for file

Additional file 2 **Amino acid alignment of selected sequence within CalS central hydrophilic loop domain**. Amino acid alignment of putative CalS5 orthologues with other selected *CalS *genes. Red boxes indicate putative functional motifs. Note that a smaller fragment was amplified from *Gnetum*. *N-glycosylation site, **Casein kinase 2 phosphorylation site, ***Protein kinase 2 phosphorylation site, ****cAMP- and cGMP-dependent phosphorylation site. Click here for file

Additional file 3 **Phylogenetic tree from central loop domains of *Arabidopsis *and *Physcomitrella CalS *genes and putative *CalS5 *orthologues**. Phylogenetic tree based on alignment of predicted polypeptides for central loop domains of known *Arabidopsis*, *Physcomitrella CalS *genes and putative *CalS *orthologues identified in this study. Click here for file

Additional file 4 **Primers used in this study**. Primers used to amplify *CalS *orthologues and conduct semi-quantitative RT-PCR. Click here for file

Additional file 5 **Sources for putative *CalS *orthologues amplified in this study**. Tissue sources and cDNA fragment sizes for putative CalS orthologues amplified from taxa in this study. Click here for file
