# Supplementary material for: Developmental evolution of flowering plant pollen tube cell walls: callose synthase (CalS) gene expression patterns
Source: EvoDevo. 2011 Jul 1;2:14. doi: 10.1186/2041-9139-2-14 (PMC3146827; doi:10.1186/2041-9139-2-14)

A

```
                        (1) 1       10        20        30        40        50        60        70        80               97
Arabidopsis CalS5       (1) MAQSSTSHDSGPQGLMRRPSRSAATTVSIEVFDHEVVPASLGTIAPILRVAAEIEHERPRVAYLCRFYAFEKAHRLDPSSGGRGVRQFKTLLFQRLE
Cabomba CalS5           (1) ----------------------------------------------------------------PLFYAFEKAHRLDPTSTGRGVRQFKTALLQRLE
Physcomitrella CalS5    (1) -MASGEGAETGSTHKPRRTSRASAVGGVTESFDSEVVPSSLAAIAPILRVANEIESSTPRVAYLCRYHAFEKAHRIDPKSSGRGVRQFKTALLQRLE
```

B

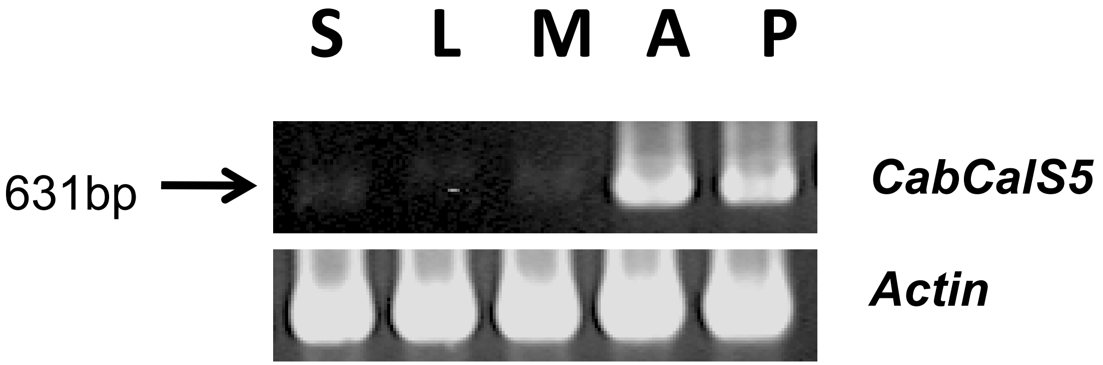

Supplement: Additional fie 1 — N-terminal alignment of CcCalS5 with AtCalS5 and PpCalS5, and CcCalS5 expression in various tissues of Cabomba caroliniana. A) Amino acid alignment showing the expected missing sequence of the N-terminal end of the CcCalS5 cDNA. B) Agarose gel showing amplified PCR products that were cloned and sequenced to confirm the presence of CcCalS5 transcript in vegetative and reproductive tissues of Cabomba. S, stem tissue, L, leaf tissue, M, meristem tissue, A, pre-dehiscent anther, P, pollen from dehiscent anther. [file 2041-9139-2-14-S1.PDF]
