# Supplementary material for: Developmental evolution of flowering plant pollen tube cell walls: callose synthase (CalS) gene expression patterns
Source: EvoDevo. 2011 Jul 1;2:14. doi: 10.1186/2041-9139-2-14 (PMC3146827; doi:10.1186/2041-9139-2-14)

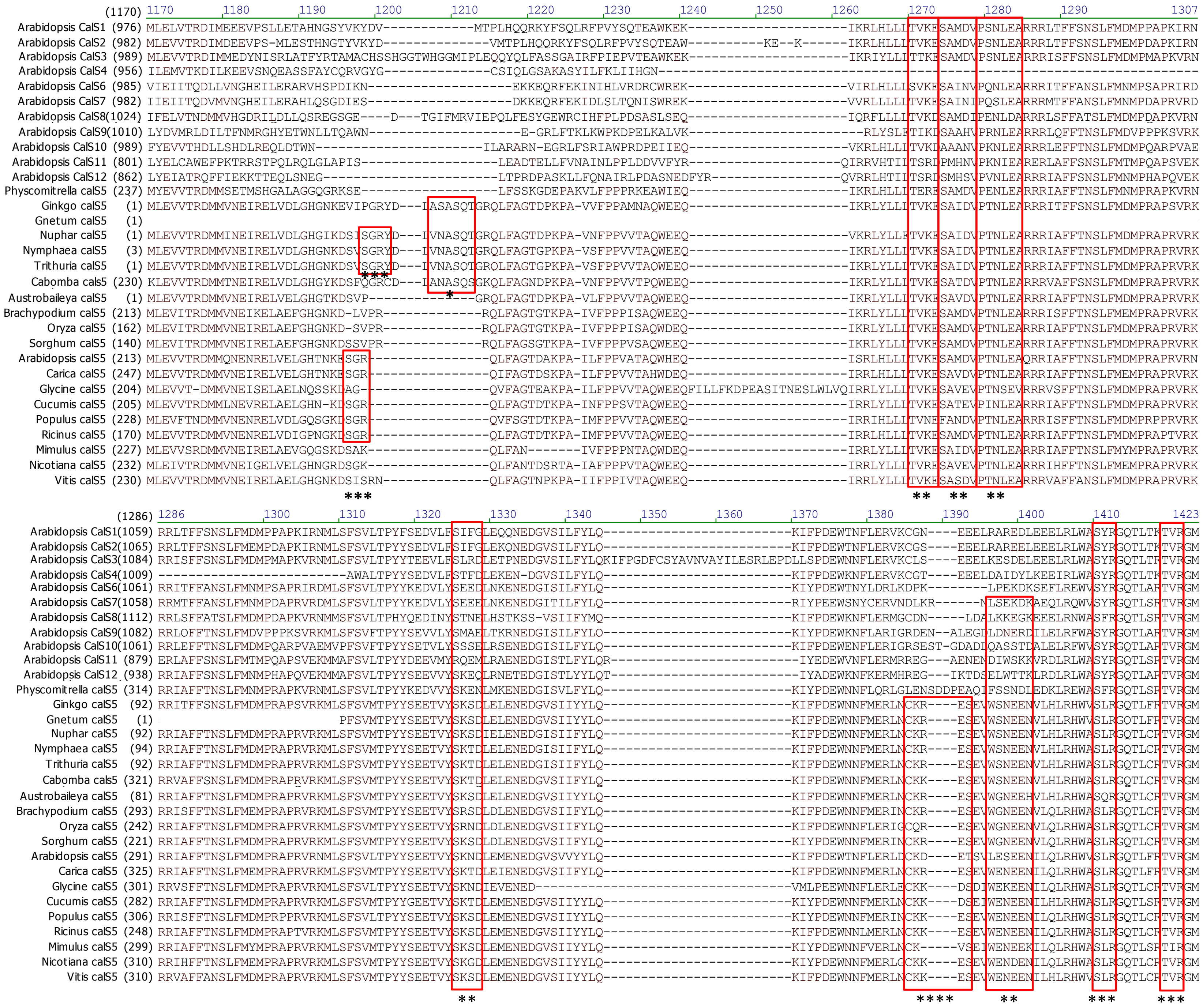

Supplement: Additional file 2 — Amino acid alignment of selected sequence within CalS central hydrophilic loop domain. Amino acid alignment of putative CalS5 orthologues with other selected CalS genes. Red boxes indicate putative functional motifs. Note that a smaller fragment was amplified from Gnetum. *N-glycosylation site, **Casein kinase 2 phosphorylation site, ***Protein kinase 2 phosphorylation site, ****cAMP- and cGMP-dependent phosphorylation site. [file 2041-9139-2-14-S2.PDF]
