# Supplementary figures and images for: Developmental evolution of flowering plant pollen tube cell walls: callose synthase (CalS) gene expression patterns
Source: EvoDevo. 2011 Jul 1;2:14. doi: 10.1186/2041-9139-2-14 (PMC3146827; doi:10.1186/2041-9139-2-14)

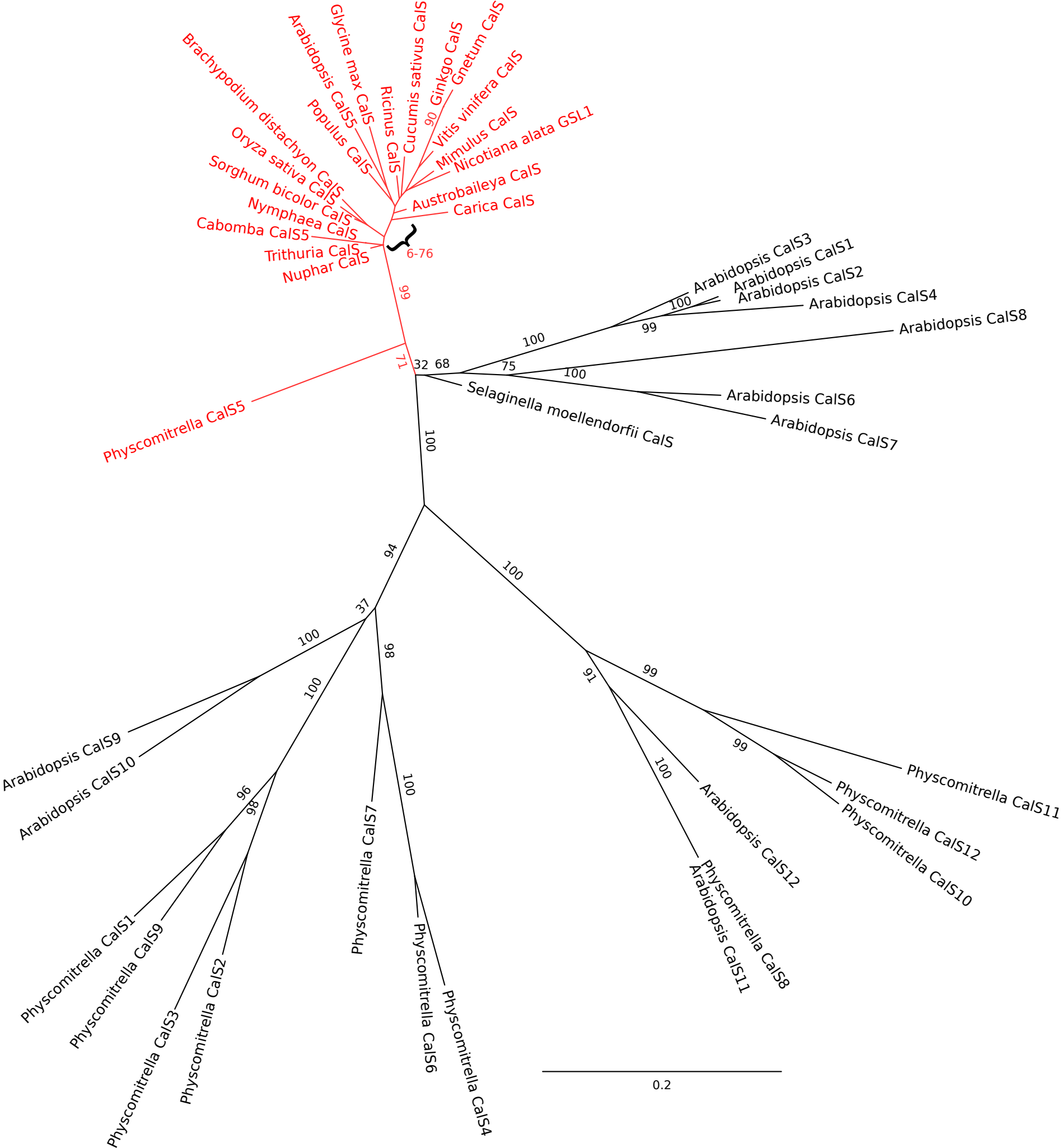

Supplement: Additional file 3 — Phylogenetic tree from central loop domains of Arabidopsis and Physcomitrella CalS genes and putative CalS5 orthologues. Phylogenetic tree based on alignment of predicted polypeptides for central loop domains of known Arabidopsis, Physcomitrella CalS genes and putative CalS orthologues identified in this study. [file 2041-9139-2-14-S3.PDF]
