# Supplementary material for: Developmental evolution of flowering plant pollen tube cell walls: callose synthase (CalS) gene expression patterns
Source: EvoDevo. 2011 Jul 1;2:14. doi: 10.1186/2041-9139-2-14 (PMC3146827; doi:10.1186/2041-9139-2-14)
Supplement: Additional file 4 — Primers used in this study. Primers used to amplify CalS orthologues and conduct semi-quantitative RT-PCR. [file 2041-9139-2-14-S4.PDF]

Additional file 4. Primers used in this study

***Nuphar CalS5* primers**

Sec16F- 5’-ATGTTAGAAGTAGTTACCCGTGACATGATG -3’

Sec17F- 5’-CAGTGTTATGACCCC -3’

Sec17R- 5’-CATCATCCCTCGGACTGT -3’

Semi-quantitative RT-PCR

*Cc631*F- 5’-ATTCATCAAGTCTTGCTGCCAG-3’

*Cc631*R- 5’-CTATTCTTGCGACCCAGAAAC-3’

FJ114840F- 5’-CATGCAAGTTGGTAAAGGTCGT-3’

AI812992R- 5’-GCAAACCCCTACTGAAGGCCT-3’

ActinF- 5’-ATGGAACTGGAATGGTCAAG-3’

ActinR- 5’-TCAAGCTCTTGCTCATAGTC-3’

***CcCalS5* Inverse PCR**

Cc5INVF- 5’-CTTTGGTCAACACCAGGTCGG-3’

Cc5INVR- 5’-GACCCGAAGAAAACGGCGCAGG-3’

5’ RACE primers

Cc515F- 5’-CGGTTCTATGCGTTTGAGAAG-3’

Cc5’innerR- 5’-TGGAGCACGTGGCATATCCAT No5’innerR-5’-CACTGAAAGACAGCATCTTACG

Cc5’outerR- 5’-TGCAAAGAGTTTGCCCTCTTA No5’outerR-5’-TCTTCATTGGACCAAACCTCAC

3’ RACE primers

Cc3’innerF- 5’-TAAGAGGGCAAACTCTTTGCA No3’innerF-5’-GTGAGGTTTGGTCCAATGAAGA

Cc3’outerF- 5’-ATGGATATGCCACGTGCTCCA No3’outerF-5’-CGTAAGATGCTGTCTTTCAGTG

3’ RACE adapter- 5’-GCGAGCACAGAATTAATACGACTCACTATAGGTTTTTTTTTTTTVN-3’
