# Supplementary material for: Developmental evolution of flowering plant pollen tube cell walls: callose synthase (CalS) gene expression patterns
Source: EvoDevo. 2011 Jul 1;2:14. doi: 10.1186/2041-9139-2-14 (PMC3146827; doi:10.1186/2041-9139-2-14)
Supplement: Additional file 5 — Sources for putative CalS orthologues amplified in this study. Tissue sources and cDNA fragment sizes for putative CalS orthologues amplified from taxa in this study. [file 2041-9139-2-14-S5.PDF]

Additional file 5 - Sources for putative *CalS* orthologues amplified in this study

Taxon	Orthologue identity	Genbank accession #	cDNA size (bp)	Tissue source(s)
Austrobaileya scandens	*CalS5*	JN021163	567	pollen; pollen tube
Trithuria austinensis	*CalS5*	JN021164	600	pollen
Nuphar advena	*CalS5*	JN021165	600	pollen; pollen tube
Nymphaea odorata	*CalS5*	JN021166	3147	pollen; pollen tube
Cabomba caroliniana	*CcCalS5*	HM590615.1	5694	pollen; stem; leaf
Ginkgo biloba	*CalS5*	JN021167	600	pollen
Gnetum gnemon	*CalS5*	JN021168	250	pollen
Pinus taeda	*PtCalS13*	JN021162	1413	pollen; pollen tube
